# Closing the access barrier for effective anti-malarials in the private sector in rural Uganda: consortium for ACT private sector subsidy (CAPSS) pilot study

**DOI:** 10.1186/1475-2875-11-356

**Published:** 2012-10-29

**Authors:** Ambrose O Talisuna, Penny Grewal Daumerie, Andrew Balyeku, Timothy Egan, Bram Piot, Renia Coghlan, Maud Lugand, Godfrey Bwire, John Bosco Rwakimari, Richard Ndyomugyenyi, Fred Kato, Maria Byangire, Paul Kagwa, Fred Sebisubi, David Nahamya, Angela Bonabana, Susan Mpanga-Mukasa, Peter Buyungo, Julius Lukwago, Allan Batte, Grace Nakanwagi, James Tibenderana, Kinny Nayer, Kishore Reddy, Nilesh Dokwal, Sylvester Rugumambaju, Saul Kidde, Jaya Banerji, George Jagoe

**Affiliations:** 1 Medicines for Malaria Venture-MMV, PO Box 1826 20, rte de Pré-Bois, Geneva 15, 1215, Switzerland; 2 Malaria Public Health & Epidemiology Cluster, University of Oxford-KEMRI-Wellcome Trust Research Programme and Worldwide Antimalarial Resistance Network-WWARN, P. O. Box 43640, Nairobi, 00 100, Kenya; 3 Uganda Ministry of Health-Ug-MoH, Po Box 7272, Kampala, Uganda; 4 Uganda National Drug Authority-NDA, Kampala, Uganda; 5 Programme for Accessible Health Communication and Education-PACE, Kampala, Uganda; 6 Malaria Consortium-MC, Kampala, Uganda; 7 Surgipharm Pharmaceuticals, Kampala, Uganda; 8 I+Solutions, Pharmaceutical Management Support, Westdam 3b, Woerden, GA, 3441, The Netherlands; 9 Management Sciences for Health-MSH, Kampala, Uganda

**Keywords:** Falciparum malaria, Artemisinin-based combination therapy, Subsidized medicines, “ACT with a leaf”, Private sector, Affordable medicines facility- malaria, Uganda

## Abstract

**Background:**

Artemisinin-based combination therapy (ACT), the treatment of choice for uncomplicated falciparum malaria, is unaffordable and generally inaccessible in the private sector, the first port of call for most malaria treatment across rural Africa. Between August 2007 and May 2010, the Uganda Ministry of Health and the Medicines for Malaria Venture conducted the Consortium for ACT Private Sector Subsidy (CAPSS) pilot study to test whether access to ACT in the private sector could be improved through the provision of a high level supply chain subsidy.

**Methods:**

Four intervention districts were purposefully selected to receive branded subsidized medicines - “ACT with a leaf”, while the fifth district acted as the control. Baseline and evaluation outlet exit surveys and retail audits were conducted at licensed and unlicensed drug outlets in the intervention and control districts. A survey-adjusted, multivariate logistic regression model was used to analyse the intervention’s impact on: ACT uptake and price; purchase of ACT within 24 hours of symptom onset; ACT availability and displacement of sub-optimal anti-malarial.

**Results:**

At baseline, ACT accounted for less than 1% of anti-malarials purchased from licensed drug shops for children less than five years old. However, at evaluation, “ACT with a leaf” accounted for 69% of anti-malarial purchased in the interventions districts. Purchase of ACT within 24 hours of symptom onset for children under five years rose from 0.8% at baseline to 26.2% (95% CI: 23.2-29.2%) at evaluation in the intervention districts. In the control district, it rose modestly from 1.8% to 5.6% (95% CI: 4.0-7.3%). The odds of purchasing ACT within 24 hours in the intervention districts compared to the control was 0.46 (95% CI: 0.08-2.68, p=0.4) at baseline and significant increased to 6.11 (95% CI: 4.32-8.62, p<0.0001) at evaluation. Children less than five years of age had “ACT with a leaf” purchased for them more often than those aged above five years. There was no evidence of price gouging.

**Conclusions:**

These data demonstrate that a supply-side subsidy and an intensive communications campaign significantly increased the uptake and use of ACT in the private sector in Uganda.

## Background

Malaria is a parasitic disease that mostly affects the poorest populations of the world, with sub-Saharan Africa experiencing the heaviest disease burden [1,2]. Early diagnosis and prompt treatment is a key component of all national malaria control strategies. In 2006, the World Health Organization (WHO) case management guidelines were revised to endorse artemisinin-based combination therapy (ACT) as the first-line treatment for uncomplicated falciparum malaria [3]. In line with WHO recommendations, Uganda’s anti-malarial treatment policy since 2006 has been ACT, specifically artemether-lumefantrine (AL), as the first-line treatment for uncomplicated falciparum malaria [4]. However, this policy shift faced major implementation challenges. First, although AL is provided free of charge through the country’s extensive network of public and not-for-profit health facilities; frequent stock-outs have severely limited its actual availability [5]. Second, ACT is highly priced in private sector outlets and there is poor availability in rural shops. However, the private sector cannot be ignored, as it is the first port of call for about 60% of Ugandans seeking treatment for fever across all socio-economic groups [6].

In response to the likelihood of an emerging threat of artemisinin resistance, the Institute of Medicine (IOM) released a 2004 expert report proposing that the price of ACT, the most effective malaria treatment, would have to be subsidized at the factory-gate to make them as affordable and as available as sub-optimal monotherapies such as chloroquine [7]. The IOM report hypothesized that this would crowd out ineffective medicines in both the public and private sectors and delay the emergence and spread of parasite resistance to the artemisinin class of drugs. The IOM proposal evolved into a new initiative aimed at closing the affordability gap - the Affordable Medicines Facility, malaria (AMFm), whose phase 1 is hosted by the Global Fund. AMFm is designed to provide a subsidy for ACT at the top of the supply chain, as well as to support a core set of interventions to ensure uptake and correct use [8,9]. However, the global debate for and against this approach is on-going [10-13]. The results of the Independent Evaluation of Phase I of the AMFm will inform the Global Fund Board’s decision in November 2012 on the future of AMFm beyond the phase 1 [14].

In 2007, the Uganda Ministry of Health (Ug-MoH) and the Medicines for Malaria Venture (MMV), in consultation with national and international stakeholders, designed the Consortium for ACT Private Sector Subsidy (CAPSS) pilot study to test the viability of the AMFm approach. The Ugandan pilot was one of two designed to assess the overall feasibility of providing affordable treatment through the private sector [15,16]. The CAPSS pilot study had a number of specific and quantifiable targets including: a) to ensure that the subsidized ACT is continuously available in at least 70% of all licensed private sector outlets - the subsidy was not sanctioned for sale in unlicensed outlets in accordance with Uganda’s policy and regulatory framework; b) to increase by 50% the number of children under five years old who have access to effective treatment within 24 hours of the onset of fever; c) to ensure that 85% of people who purchase the ACT comply with the recommended treatment schedules and d) to achieve 40% market share of all anti-malarials purchased at licensed drug shops.

Here the findings from the CAPSS pilot study are reported, that demonstrate that the AMFm strategy is feasible and can improve access to life saving medicines in the private sector in rural Uganda.

## Methods

### Pilot study design

**Figure 1:**
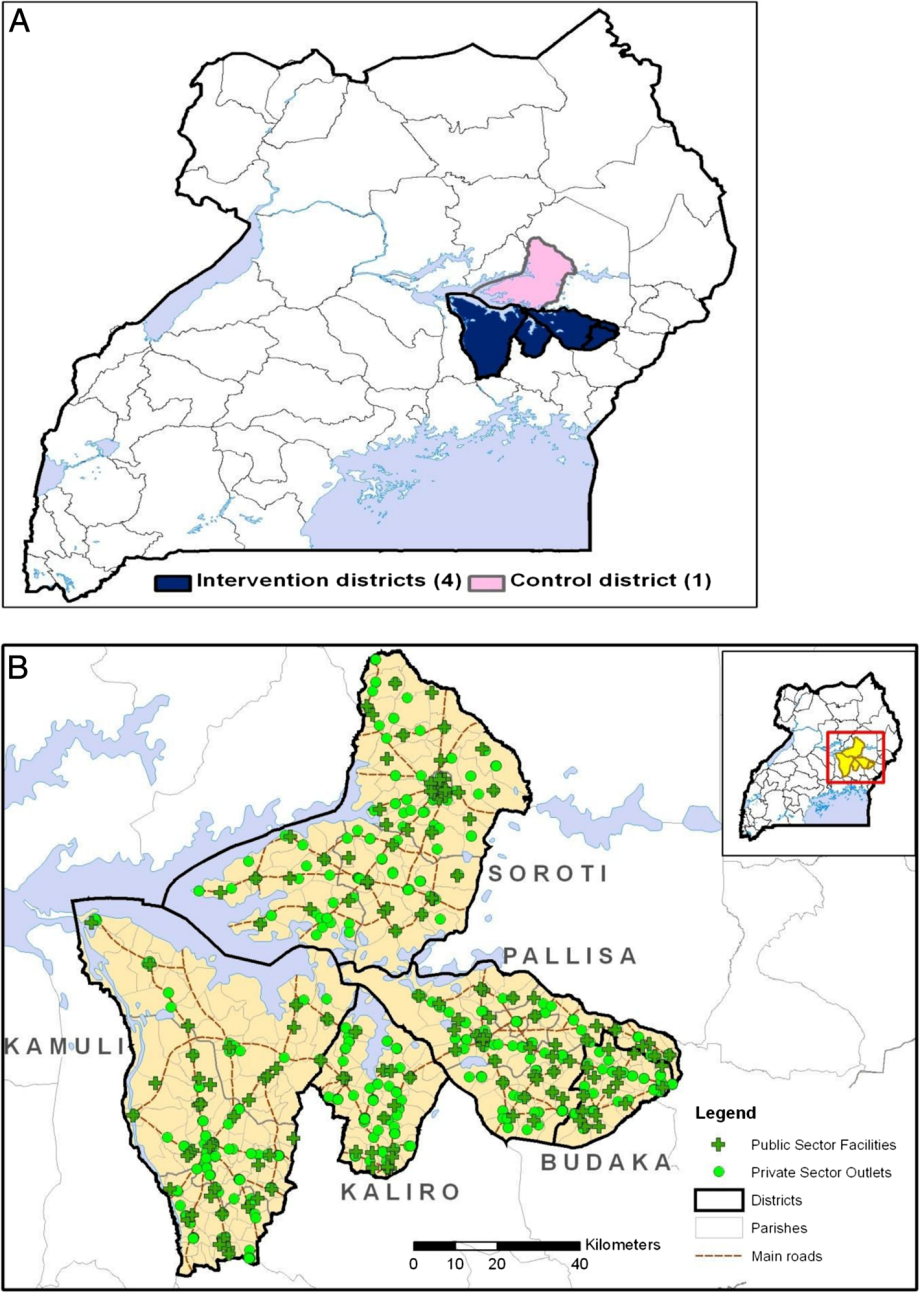
**A Map of Uganda showing the location of the intervention and control districts.B** Geographical distribution of public and private health drug outlets in the intervention and control districts.

Four contiguous intervention pilot districts (Kamuli, Kaliro, Pallisa, and Budaka) were selected to receive the subsidized medicines, while the fifth district (Soroti) acted as the control. To limit leakage of the intervention to the control area, the control and intervention areas had two intervening buffer districts (Bukedea and Kumi) or a lake between them (Figure 1A). The pilot districts had a total of 104 public health facilities and over 750 private sector outlets, predominantly drug shops (licensed and unlicensed). The private sector was also more widely distributed across the districts (Figure 1B). All the five districts have perennially high malaria transmission, with estimated parasite prevalence above 60% in school-going children [un-published MMV-Ug-MoH report]. At the time of study initiation, the overall population of the intervention districts was 1.4 million people [Uganda Bureau of Statics, Population Census Projections].

### Baseline surveys

Prior to the pilot study, in July 2007, the Ug-MoH and MMV carried out a two-part baseline household survey in order to fully understand the supply and demand for anti-malarials [6,17]. ^.^The baseline household survey provided a comprehensive picture of malaria treatment, which subsequently shaped the pilot’s design. One of the baseline household survey’s findings was that access to effective treatment was extremely low. In 2007, in the most vulnerable age group, of children under the age of five years, only 3% of those contracting a fever received effective treatment (ACT) within 24 hours of symptom onset. This figure rose to just 4% after 48 hours of symptoms onset [6]. The baseline household surveys also demonstrated that 60–70% of people obtained anti-malarial treatment from private sector drug shops [6]. The private sector was dominant in part because private outlets are more numerous and widespread and in part because drug shops were better stocked and open for longer hours. Further, stock-outs of all anti-malarial medicines were very frequent in the public sector facilities [5]. In addition, the baseline household surveys highlighted price differences that may play a key role in determining which medicines caregivers use to treat malaria. The price range for a full dose of ACT, in 2007, was Ugandan shillings (UGX) 9,000-20,000 (USD 5.40-12.00), while that for a full course of chloroquine in a drug shop was UGX 200–500 (USD 0.12-0.30) [17]. The predominant proportion of Ugandans live on less than USD 1.25 a day, suggesting that price matters greatly.

### A comprehensive and multi-pronged intervention

The CAPSS Uganda pilot study was designed in line with the AMFm approach of providing subsidized medicines in combination with supporting interventions, including provider training and demand generation. Several consultative meetings were held in Uganda to design the intervention and build on existing best practices. The pilot study was approved by all the relevant competent bodies of the Ug-MoH and ethical approval was obtained from the competent research ethics committee. During the baseline and periodic evaluation conducted through outlet client exit surveys, each respondent provided informed consent before being interviewed.

### Pilot study management structure to ensure proper governance

The top most level of the CAPSS pilot study management structure comprised the project management team consisting of representatives from the Ug-MoH, the National Drug Authority-NDA and MMV. The implementation team, responsible for managing the day-to-day aspects of the pilot study, included representatives from the lead CAPSS partners - Programme for Accessible Health Communication and Education (PACE), Surgipharm Pharmaceuticals, Malaria Consortium-MC, Ug-MoH, NDA, MMV and the district health officers of the pilot districts.

### Aligning the intervention to the policy and regulatory framework

Prior to the pilot, AL was regulated as a prescription-only medicine. In order to facilitate the CAPSS intervention, the Uganda NDA rescheduled AL so that it could be provided over the counter within the intervention area.

### Repackaging and branding to ensure the subsidized product stands out

**Figure 2:**
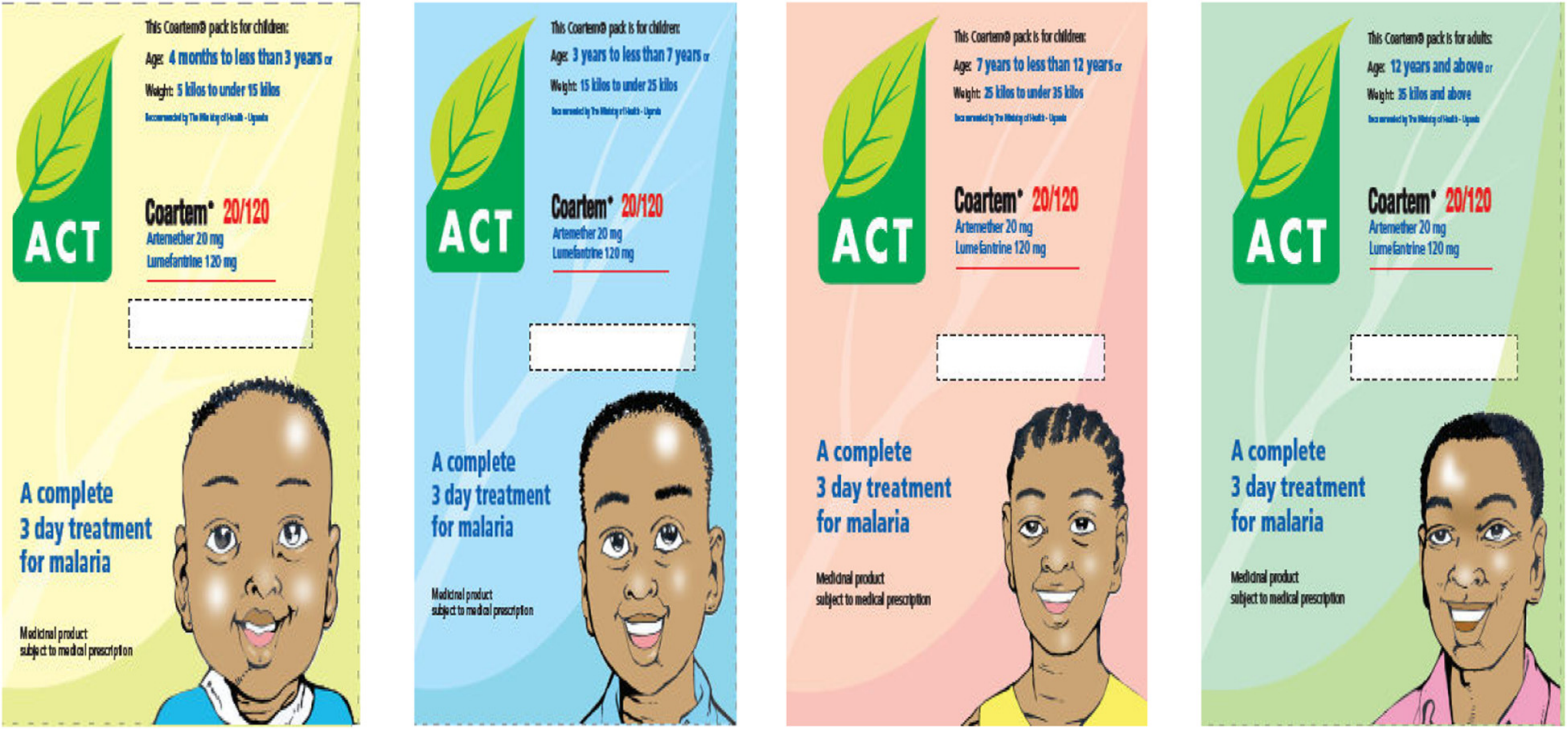
Branded Coartem - “ACT with a leaf” in the four age packs.

New packaging was designed for the subsidized AL (Coartem®) to differentiate the subsidized product from the public sector offering. The packaging was also designed to facilitate the correct use of the product, incorporating illustrated instructions on drug usage. AL was packaged in four colour-coded packs for four different age bands. As part of the repackaging exercise the subsidized AL was branded “ACT with a leaf” to distinguish it from all other forms of ACT and anti-malarials (Figure 2). The distinctive branding of “ACT with a leaf” provided consumers with the instant recognition that they were purchasing a high quality and effective anti-malarial at an affordable price.

### Communications programme to take the message to caregivers

A communications campaign rooted in the caregivers’ knowledge, understanding and perception of the recommended health behaviours was instituted. The campaign was comprehensive, including: community mobilization, community events, radio spots and talk shows, posters, point of sales advertising, songs and community launch events, and was supported by high level ministry of health and district officials. The wide array of communication activities generated significant brand awareness within a short time.

### Supply chain management to get the subsidized product on the shelf

The quantification of the amount of Coartem required for the pilot study was based on estimates of the number of fever episodes per age group using available epidemiological data, the likely source of treatment based on the findings from the household survey, and a buffer for leakage. A third party was responsible for the importation of the ACT and its storage, as well as for overseeing the repackaging, and the distribution of “ACT with a leaf” to licensed outlets within the intervention districts. In line with the AMFm design, the CAPSS pilot sought to replicate the existing supply chain. However, recognizing that all the intervention districts are very rural and lack a wholesaler within their boundaries, the pilot improved the distribution chain by ensuring that licensed drugs shops directly received their supplies of “ACT with a leaf” from the distributor or sub-distributor. Two different compensation models to cover the additional costs of direct distribution were tested. The costs of weekly distribution to outlets in Pallisa and Budaka were covered directly by MMV, whereas those for Kamuli and Kaliro came out of the distributor’s margins.

### Pricing to ensure affordability

In line with the AMFm concept, MMV subsidized AL by 95%. The import prices for the subsidized product for each weight band, corresponding to the four different coloured age packs, was USD 0.05, 0.10, 0.15, 0.20, respectively. The final price was calculated in relation to the market prices for other anti-malarials, taking into consideration standard commercial margins. A maximum recommended retail price (MRRP) for each age pack was printed on the product and the study ensured that the price per tablet in each pack was identical, thereby removing any potential commercial interest in cutting up the packs. The final MRRP per age pack ranged from UGX 200 to UGX 800 (USD 0.10 to USD 0.40).

### Training to ensure drug shop attendants have the requisite knowledge

The training of drug shop attendants was done to ensure the correct dispensing and use of “ACT with a leaf”. Prior to the launch of the pilot study, at least two drug shop attendants from each licensed outlet participated in comprehensive training. This covered malaria case management and product-specific knowledge, including safety monitoring and supply logistics. Refresher training sessions were also provided during the regular drug distribution circuits.

### Monitoring to keep track of impact

In September 2008, a baseline client exit survey (n=1162) was administered to clients exiting all the private drug shops/private clinics/pharmacies (hereafter referred to as “outlets”) in the study districts. These surveys were conducted by trained interviewers from the research organization - Synovate Kenya and Uganda. The interviewers were instructed to reach every outlet in all the study districts during a period of approximately two weeks and to administer interviews to exiting clients who were purchasing any medicine(s) for the treatment of febrile illness. Following the baseline survey, CAPSS initiated the ACT subsidy for the intervention districts and four subsequent rounds of monitoring of cross-sectional surveys were administered in the same manner as the baseline survey, with the final round occurring during the period 20 April - 11 May, 2010 (Baseline: n=1,162; round 1: n=1044; round 2: n=1794; round 3: n=1976 and final round 4: n=5181). A minimum number of 5 interviews per outlet were respected in the final survey round, resulting in a significantly higher sample size. For this paper, the baseline and final evaluation survey data were the primary focus. However, some analysis has been included for the other survey rounds to provide a comprehensive picture. During each survey round, we also tracked stocking and purchasing behaviour of “ACT with a leaf” other ACT and other anti-malarials at outlets and stocking of all anti-malarials at public health facilities.

### Data management and analysis

The outlet survey, retail and public health facility audit data at baseline and follow up were uploaded into a secure Microsoft Access © database and were converted to an SPSS 18.1® database and Stata version 12.0® Stata Corp Texas USA for statistical analysis. Observations were categorized according to socioeconomic status (SES) quintiles based on scores generated by a factor analysis of binary SES-indicator variables. Based on the data-collection methods, a survey-adjusted logistic regression model was used. The outlets were treated as the population sampling units within five strata - the five pilot districts. As the sampling method was a best attempt at an outlet census, no weighting was applied. To minimize data correlation (i.e. information on multiple febrile patients), one observation per respondent was randomly selected for inclusion in the analysis. Because the intervention was administered at the district level, an intention-to-treat (ITT) analysis was performed despite the fact that unlicensed outlets in the intervention districts were not offered the subsidized ACT. Univariate, survey-adjusted logistic regression models were constructed to examine the intervention effect sizes. Finally, the remaining covariates were each treated as the outcome in a univariate, survey-adjusted logistic regression model with intervention status as the exposure variable. In the step-wise, multivariate logistic regression, covariates were included in descending order of the strength of evidence supporting their association with intervention status - smallest to largest Wald Test p-value. For covariates with indiscernibly small p-values, the variables possessing the most dramatic odds ratios (ORs) between categories were prioritized for inclusion in the multivariate model. Additionally, all variables for which the p-value of the association with the intervention status was ≥0.5 were excluded as potential confounders. If inclusion of a covariate in the multivariate logistic regression model produced a delta of >10% between the adjusted and unadjusted ORs for the primary exposure, it was deemed a confounder and kept in the model unless subsequent tests for heterogeneity produced sufficient evidence (p<0.05) that it was an effect modifier in which case the degree of interaction was explored. Once all identified confounders were included in the model, a separate model was fitted with an interaction term between intervention status and each variable with a plausible, independent correlation with either the ACT uptake or intervention status. Adjusted Wald tests of these interaction parameters were conducted to determine whether any variables interacted with the effect of intervention status on ACT uptake, with a significance cut-off of p<0.05. If evidence of multiple effect modifiers emerged from these tests, the stratum-specific ORs for each interacting variable were presented separately. Due to the length of the survey and the large number of respondents, missing values were inevitable. In the event that a variable included in a survey-adjusted model had missing values, listwise deletion of such observations was performed. Further, in the event that a category within a given variable possessed so few affirmative responses to the extent that it perfectly predicted outcome success or failure, such observations were likewise excluded. Visual analysis was conducted to assist with the interpretation of availability findings using geographic information systems with Esri ArcGIS software.

## Results

### Primary analysis: final evaluation round

Of the 5,643 observations collected in the final evaluation survey, 5,181 observations resulting from visits to 783 outlets were included in the analysis. The median age of respondents was 28 years (range: 10–74 years), and 50.1% were male. Among patients, the median age was 15 years (range <1-92 years) and 54.2% were male. Interviews in intervention districts accounted for 77% of the observations, taking place at 600 outlets.

82% of licensed outlets in the intervention districts carried stocks of the subsidized ACT at the final round of data collection. Among all respondents, 21% had purchased some form of ACT. Survey-adjusted, one-way frequency estimates and corresponding 95% confidence intervals (95% CI) are provided in Table 1. After adjusting for the survey structure, 77% (95% CI: 75.8-78.8%) of respondents indicated that the outlet they were exiting was the first place they had been to seek treatment. Of the 23% who reported having first sought treatment elsewhere, 68% (95% CI: 64.9-71.3%) had initially visited a public health facility. The most common reason cited for choosing the outlet at which the respondent was interviewed was proximity (32%; 95% CI: 30.3-34.0%), followed by receiving a recommendation or referral (18%; 95% CI: 16.7-19.2%).

**Table 1:** Survey-adjusted, one-way frequencies of select variables

**Characteristic**	**Value**	**N**	**%**	**95% CI**
Antimalarial category purchased	Chloroquine	468	9.0	7.9-10.2
	ACT	1,109	21.4	19.1-23.8
	Quinine	2,356	45.5	43.5-47.6
	SP	661	12.8	11.6-14.0
	Other antimalarial	110	2.1	1.6-2.7
	Non-antimalarial	469	9.1	8.0-10.1
Had the shop at which the interview took place been supplied with subsidized ACT according to Surgipharm data?	Not supplied	4,146	80.1	77.3-83.0
	Supplied	1,027	19.9	17.0-22.7
Age of the patient	<5 years	1,726	33.4	32.0-34.8
	≥5 years	3,442	66.6	65.2-68.0
Age of the respondent	<20 years	767	15.0	13.9-16.1
	20-29 years	2,156	41.7	40.4-43.1
	30-39 years	1,502	29.1	27.9-30.2
	≥40 years	732	14.2	13.2-15.1
Gender of the patient	Female	2,370	45.8	44.6-47.0
	Male	2,803	54.2	53.0-55.4
Gender of the respondent	Female	2,581	49.9	48.4-51.3
	Male	2,592	50.1	48.7-51.5
How long ago did symptoms begin?	<24 hours	2,862	57.0	54.9-59.0
	24-48 hours	1,551	30.9	29.2-32.5
	>48 hours	612	12.2	11.0-13.4
How long of a delay was there between onset of patient’s symptoms and initiation of any treatment?	<24 hours	3,221	64.0	61.9-66.1
	24-48 hours	1,438	28.6	26.7-30.4
	>48 hours	377	7.5	6.6-8.4
How long does it take the respondent to walk to the outlet at which the interview took place?	<15 minutes	2,223	43.1	41.1-45.1
	15-29 minutes	1,898	36.8	35.1-38.5
	30-59 minutes	860	16.7	15.2-18.1
	1-1.9 hours	150	2.9	2.3-3.5
	≥2 hours	31	0.6	0.3-0.8
How long does it take the respondent to walk to the nearest public health facility?	<15 minutes	685	13.3	11.8-14.8
	15-29 minutes	1,471	28.6	26.6-30.5
	30-59 minutes	2,014	39.1	36.9-41.4
	1-1.9 hours	777	15.1	13.4-16.7
	≥2 hours	199	3.9	3.0-4.8
What is the primary reason that the respondent selected this medicine?	Used it before	2,550	49.3	47.5-51.1
	Recommended by health worker/seller	2,137	41.3	39.6-43.1
	Only medicine available	210	4.1	3.2-4.9
	Price	184	3.6	2.9-4.2
	Other	91	1.8	1.4-2.1

Post survey-adjustment, it was found that 26.2% (95% CI: 23.2-29.2%) of respondents in the intervention districts purchased ACT compared to 5.6% (95% CI: 4.0-7.3%) in the control district. The survey-adjusted, univariate logistic model indicated that patients in the intervention districts had a six-fold increase in ACT use relative to the control district (95% CI: 4.22-8.44, adjusted Wald test p<0.001) (Table 2). The investigation of potential confounders did not yield any variables that sufficiently impacted the unadjusted OR. However, tests for heterogeneity indicated evidence of effect modification by two variables, separately: “age of the patient” (p=0.02) and “highest school level completed by the respondent” (p=0.003). There was only weak evidence (p=0.08) of a three-way interaction between these variables and intervention status (Table 3). The observed ORs suggest that the effect of intervention status on ACT uptake diminishes with patient age. The stratum specific ORs also indicate that the effect of intervention status on ACT uptake tends to be more pronounced among those with lower education than among the better educated (Table 3).

**Table 2:** Univariate analysis of antimalarial uptake by intervention status

**Outcome**	**Intervention status**	**OR**	**95% CI**	**p-value**
ACT use	Control	1	-	p<0.0001
	Intervention	5.97	4.22-8.44	
Quinine use	Control	1	-	p<0.0001
	Intervention	0.25	0.2-0.3	
SP use	Control	1	-	p=0.004
	Intervention	1.49	1.14-1.95	
Chloroquine use	Control	1	-	p=0.3
	Intervention	1.16	0.86-1.59	

**Table 3:** Stratum specific ORs comparing ACT use between intervention/control districts

**Effect modifier**	**Value**	**Intervention status**	**OR**	**95% CI**	**p-value**
Age of patient	<1 years	Control	1	-	p<0.001
		Intervention	8.48	5.25-13.67	
	per each additional 1 year	Control	1	-	p=0.02
		Intervention	0.98	0.96-1.00	
Respondent’s highest school level completed	None	Control	1	-	p<0.001
		Intervention	10.20	3.11-33.41	
	Primary	Control	1	-	p<0.001
		Intervention	6.49	3.84-10.98	
	O Level	Control	1	-	p<0.001
		Intervention	8.49	5.05-14.29	
	A Level	Control	1	-	p<0.001
		Intervention	2.88	1.64-5.03	
	University	Control	1	-	p=0.003
		Intervention	4.84	1.71-13.66	

### Improved access to treatment

**Figure 3:**
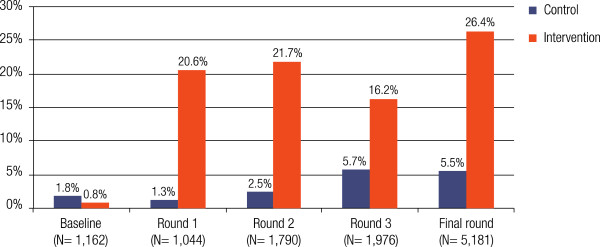
Purchase of ACT within 24 hours of symptom onset at baseline, monitoring rounds (1, 2 and 3) and final survey round.

There was higher access to treatment within 24 hours of fever onset in the intervention districts compared to the control district. At baseline, only 0.8% of respondents purchased an ACT within 24 hours of fever onset in the intervention districts. This figure rose to 26.2% in final survey round. In contrast, the equivalent figure in the control district rose from 1.8% at baseline and to a modest 5.5% at the final survey round (Figure 3). The odds of purchasing treatment within 24 hours in the intervention compared to the control districts at baseline were 0.46 (95% CI: 0.08-2.68, p=0.4) and rose to 19.2 (95% CI 6.08-60.2), 11.02 (95% CI: 5.41-22.02) at survey rounds 1 and 2 and stabilized at 6.11 (95% CI: 4.32-8.62, p<0.0001) at survey round 4 (Table 4).

**Table 4:** Odds of accessing ACTs in the intervention vs. control districts at baseline, monitoring rounds (1, 2, 3) and final evaluation survey

**Survey wave**	**Intervention status**	**Adjusted OR**	**95% CI**	**P value**
Baseline (n=1162)	Control	1	Reference	
	Intervention	0.46	0.08-2.68	0.4
Round 1 (n=1044)	Control	1	Reference	
	Intervention	19.2	6.08-60.2	<0.0001
Round 2 (n=1794)	Control	1	Reference	
	Intervention	11.04	5.41-22.52	<0.0001
Round 3 (n=1976)	Control	1	Reference	
	Intervention	3.29	1.76-6.13	0.0002
Final round (n=5173)	Control Intervention	1 6.11	Reference 4.32-8.62	<0.0001

### Sub-analysis: prompt treatment of patients less than 5 years old

A sub-analysis, focusing on patients less than five years old, included 1,726 observations in the final evaluation survey. There was very strong evidence that children under five years old in the intervention districts experienced 10 times the odds (95% CI: 4.96-18.86, adjusted Wald test p<0.001) of receiving ACT within 24 hours of symptom onset compared to children in the control district. The survey-adjusted proportion of children receiving prompt and effective treatment in the intervention district was 18% (95% CI: 14.9-20.8%) compared to 2% (95% CI: 0.8-3.6) in the control district.

### Sub-analysis: predictors of ACT uptake in intervention districts

This sub-analysis of intervention districts included 3,965 observations. Six variables were associated with uptake of ACT in these districts (Table 5). Encouragingly, ACT purchasers exhibited three times the odds of citing “drug price” as the primary reason for purchasing their medicine relative to non-ACT purchasers (6% vs. 2.5%; p<0.001). Further, the proportion of ACT purchasers indicating that they felt the medicine they purchased was affordable or very affordable was 61% (95% CI: 56.8-64.3%) and 25% (95% CI: 20.9-28.7%), respectively. In contrast, the proportion of non-ACT users saying the same was 35% (95% CI: 33.0-37.4%) and 8% (95% CI: 6.6-9.1%). There was insufficient evidence that this perception of ACT affordability was heterogeneous by SES level (p=0.08). Moreover, children under five years old were more likely to have ACT purchased on their behalf than patients aged five years or older (29% vs. 25%; OR: 1.38, 95% CI: 1.17-1.64; p<0.001). However, members of the highest SES stratum exhibited 2.4 times (95% CI: 1.72-3.35; p<0.001) the odds of ACT use compared to the lowest stratum. Additionally, respondents purchasing medicine 24 to 48 hours after the onset of the patient’s fever were more likely to buy ACT than those purchasing drugs within 24 hours (30% vs. 25%; OR: 1.37; 95% CI: 1.10-1.70; p=0.004) (Table 5).

**Table 5:** Characteristics associated with ACT use in intervention districts

**Characteristic**	**Value**	**OR**	**95% CI**	**p-value**
“Price” cited as the primary reason for choosing medicine by respondent	No	1	-	p<0.001
	Yes	2.56	1.67-3.94	
Socioeconomic status quintile (1–5: lowest to highest)	1	1	-	p<0.001
	2	1.51	1.13-2.01	
	3	1.65	1.23-2.21	
	4	1.84	1.35-2.51	
	5	2.40	1.72-3.35	
Age group of patient (relative to 5 years)	≥5 years	1	-	p<0.001
	<5 years	1.38	1.17-1.64	
“Only medicine available” cited as the primary reason for choosing medicine by respondent	No	1	-	p=0.001
	Yes	0.44	0.27-0.71	
Time since onset of patient’s malaria symptoms*	<24 hours	1	-	p=0.01
	24-48 hours	1.37	1.10-1.70	
	>48 hours	1.10	0.76-1.59	
Length of time it takes the respondent to walk to the nearest public health facility	<15 minutes	1	-	p=0.03
	15-29 minutes	0.82	0.62-1.08	
	30-59 minutes	0.59	0.42-0.83	
	1-1.9 hours	0.71	0.46-1.09	
	≥2 hours	0.90	0.46-1.77	

* Time since symptom-onset” and “whether the outlet was the respondent’s first stop to seek treatment” appear to be co-linear. This former variable was chosen for the model as it is more relevant to the objectives of CAPSS.

### Secondary analysis: sub-optimal anti-malarial use at baseline and final survey round

Of 1,173 observations in the baseline data, 1,168 were subjected to analysis. This investigation found that there were no notable associations between prospective intervention status and use of SP or ACT. However, this analysis did uncover weak but not significant evidence that the intervention districts experienced less quinine use relative to the control district (37% vs. 44%; OR: 0.76; 95% CI: 0.53-1.08, p=0.1).

### ACT with a leaf uptake in the intervention districts

**Figure 4:**
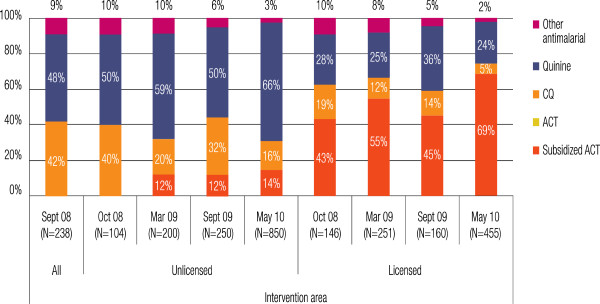
Market share of “ACT with a leaf” in the intervention districts at baseline, monitoring and final evaluation survey.

By the end of the pilot study, “ACT with a leaf” accounted for 69% of all anti-malarials purchased at licensed drug shops to treat malaria in the under-five year old age group in the four intervention districts (Figure 4). In line with national regulations, “ACT with a leaf” was not sold to unlicensed drug shops; however, the product leaked to some outlets, resulting in 14% of the caregivers of children visiting unlicensed outlets receiving “ACT with a leaf”. The share of subsidized ACT among all anti-malarials purchased dropped in September 2009 as demand outstripped supplies, resulting in stock-outs. This was itself largely due to the knock-on effect of protracted public sector ACT stock-outs, resulting in virtually all access to ACT in the intervention areas being through the private sector. This also increased the share of quinine sales from licensed drug shops and chloroquine sales in unlicensed outlets.

### Low price made “ACT with a leaf” the medicine of choice

**Figure 5:**
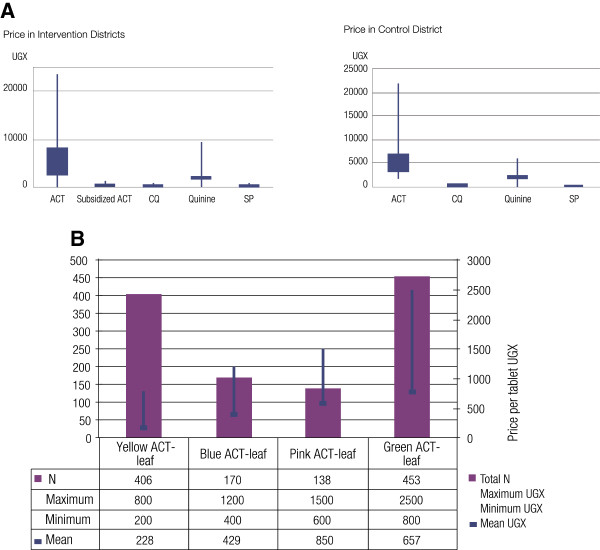
**A. Price of subsidized ACT in comparison to chloroquine.B**. Price of subsidized ACT largely respected, rendering it affordable.

The subsidy brought the price of ACT in line with that of chloroquine and a full course of a child’s treatment was lower than that for quinine (Figure 5A). The subsidy along with a good communication campiaign was highly successful in ensuring that the maximum recommended retail price (MRRP per tablet: UGX 33) was respected, particularly in licensed outlets, with the mean price varying within a 10 percent band (Figure 5B).

### Most children received the right dose

**Figure 6:**
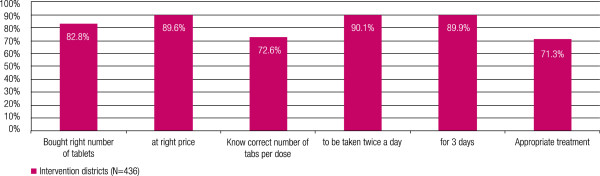
Appropriateness of treatment doses purchased across the intervention districts.

Over 80% of caregivers of children under five (except in Pallisa) purchased the correct number of tablets at the right price. Knowledge of the correct dosing varied: about 70% of caregivers knew the correct number of tablets per dose and over 80% knew that the dose should be given twice a day for three days. The level of knowledge in Budaka was higher than the other districts. The composite indicator across all intervention districts for the appropriateness of the treatment administered was 71%, with the lowest score of 65% in Pallisa and the highest of 89% in Budaka (Figure 6).

## Discussion

The CAPSS pilot study was successful in demonstrating that a subsidized product coupled with an intensive communication campaign leads to increased purchase and use of ACT in the private sector. There was a six-fold increase in the number of people (all age groups) and a ten-fold increase in children under five years old purchasing effective malaria treatment within 24 hours of the onset of fever. Further, over 70% of the caregivers who purchased an ACT complied with recommended treatment schedules. All the objectives set at the outset were thus achieved.

The CAPSS pilot study validates the hypothesis that affordable treatment drives availability and uptake, thereby displacing ineffective treatments, and provides evidence to support the AMFm concept. The preliminary findings from the CAPSS pilot study were shared with the AMFm task force in 2010 and provided the rationale for using the universal logo for all AMFm products [15] - the CAPSS logo of “ACT with a leaf” was chosen as the AMFm product logo (“ACTm with a leaf”).

We believe the effect sizes we observed between the intervention and control areas are statistically modest compared to what could have reasonably been expected. There are two reasons for this. First, the overall ACT availability in public health facilities in the control district (Soroti) improved greatly, with three-quarters of facilities having ACT in stock in May 2010 [Uganda HMIS reports]. The contextual information suggests that there was better availability of ACT in the public sector in the control district because of improvements that had been made in the procurement and distribution system, based on a push instead of a pull system for the lower-level health units. However, this was not the case in the intervention districts. Consequently, slightly more than half of the public health facilities in Pallisa and Kaliro had no public sector ACT packs in stock at the time of the surveys; and the situation in Budaka was distressingly inadequate, with three quarters of public sector facilities having no ACT in stock. Second, the intervention made by another group (Living Goods) to improve access to ACT at community level in the control district had an impact on ACT supply there. These findings make us believe that the effect differences could have been bigger had these developments not taken place or had the public sector supply chain not failed in the intervention districts.

The private sector’s role in ensuring access to medicines is largely complementary to that of the public sector: the nature of this role is determined primarily by the level of availability in public sector outlets. When there are stock-outs in the public sector, the private sector’s role inevitably shifts from a complementary one to primary. Whether the private sector played a complementary or primary role therefore varied greatly during the different rounds of data collection. For example, in Kaliro, Pallisa and Budaka, access to ACT was largely assured through the availability of the subsidized ACT in the private sector, because stock-outs in the public sector were very frequent. However, in Kamuli, private sector availability complemented existing public sector availability. ACT access through the private sector was very low in the control district. Based on these findings, we believe that moving forward; the AMFm strategy should be tailored to the local context [13]. There are settings where strengthening the public sector can achieve substantial improvements in access to malaria treatment. However, in settings like Uganda, strengthening the public sector without providing complementary support from the private sector is very unlikely to achieve the WHO Global Malaria Action Plan (GMAP) case management access target that 80% of patients should receive treatment within 24 hours of symptom onset [18].

While the CAPPS pilot study was very successful, there are several challenges that need to be addressed to ensure adequate availability in poor and remote rural areas. This requires a number of specific actions, such as incentivising suppliers to deliver medicines to such areas, or strengthening community based systems, such as that of Uganda’s village health teams. Ensuring there are sufficient licensed outlets to meet demand is also critical. In the more developed areas of Uganda, where private businesses flourish, a sufficiently large number of private outlets are able to achieve the required licensing standards. However, in other less developed areas, where there are currently few or no private licensed outlets, there is insufficient coverage. In addition, the outlets that do exist in these areas are often severely constrained, since many of the smaller outlets rely on the immediate cash flow from sales to pay for their next orders. These cash flow constraints result in the smaller outlets placing very small orders with the distributors, since they cannot afford to tie up significant amounts of money in stock. Prices in the more remote rural areas are often even higher than in urban/suburban centres, largely due to the additional costs of distribution. Enhancing access to ACTs in poorer, more remote areas requires overcoming these distribution challenges.

A second challenge is the difficulty of maintaining the maximum recommended retail price in the face of currency exchange fluctuations. The Ugandan Shilling faced ongoing devaluation during the course of this study. This resulted in the importers of “ACT with a leaf” facing higher landed costs, costs which are normally passed on to the consumers in terms of higher prices. Moreover, national drug regulatory agencies in many countries, particularly in East Africa, do not attempt to control market prices in view of price liberalization policies. Rather than imposing a maximum price, therefore, a more pragmatic approach might be to issue a “recommended retail price range” (RRPR). This would be effective in setting price expectations. The maintenance of the ACT price is also subject to the availability of the continuing subsidy; this price is consequently vulnerable to changes in the global funding landscape.

A third challenge, applying to both the public and private sectors, is the need to avoid stock-outs. Ensuring uninterrupted availability of ACT is critical to guaranteeing access to effective treatment. In this regard, the two sectors can work synergistically to improve access to treatment. A concerted effort needs to be made to ensure that more caregivers of children under five seek treatment within 24 hours of the onset of fever and have access to ACT. The introduction of rapid diagnostic tests in the public sector and subsequently in the private sector could help ensure targeting of ACT. Such an approach would ensure that patients quickly get access to the correct treatment for malaria based on parasite-based diagnosis as per current WHO recommendations [19].

The results of the independent evaluation of the AMFm from Uganda validate the CAPSS findings of affordability driving stocking and uptake as availability of quality assured ACTs (AQ-ACT) across all outlets increased from 21% at baseline to 67% at endline [14]. The recommended retail price (RRP) however of USD 0.47 for an adult course of treatment was not adhered to; the median price at the endline survey was USD 1.96. The independent evaluation suggests that this may be influenced by the absence of supporting interventions before the final round of data collection due to delays in the disbursement of the Global Fund grant and the fact that only 10% of respondents knew that there was a RRP and only 5% knew the level.

Certain limitations in the design of this study need to be noted. First, the districts were purposefully selected and only one district was used as a control. This could have increased the potential for discordance between the populations included in the study. Some discordance was observed between the intervention and control districts at baseline in terms of drug consumption habits. Fortunately, the observed disparities did not include the use of ACT. Generating survey-adjusted outputs was intended to provide a more reasonable range of likely values that accounted for this prior to executing tests of significance. Second, only respondents leaving drug outlets at specific times and who consented to the interview were analysed. This could lead to both a selection and response bias. Different groups are likely to visit drug stores at different times during the day. As an example, people who are employed full-time may be more likely to visit an outlet at night. Also, we did not collect detailed information about potential respondents who refused to be interviewed. It is possible that their characteristics differed from the study sample. However, the rate of refusal was generally small (not exceeding 10%) and any effect due to refusal probably did not impact significantly on the outcome measures. Further, the wide range of times at which interviews were conducted, in combination with survey-adjustment, minimized the influence of these possible biases. Finally, a possible observer bias could arise from the fact that the interviewers were not blinded to the intervention status of the respondents. To mitigate this risk, a week long training session was administered prior to each round of data collection to instil strict processes for conducting interviews and to prevent - to the extent possible - deviation from the script. It is unlikely that this lack of blinding could influence the primary outcome in this study, as it was obligatory for the interviewer to observe and record the details of the actual medicine purchased.

## Conclusion

These findings provide evidence that a subsidy high up in the supply chain coupled with an intensive communication campaign leads to increased ACT uptake, making a compelling case for AMFm in Uganda and potentially other African settings with similar private sector use. The approach will need to be customized by country to respond to specific national access challenges. The approach adopted has been effective in extending uptake in the most vulnerable category, the youngest segment of the population. Further, it is reassuring that customers purchase the right dose, consistent with observations reported elsewhere in Uganda [20].

All of the CAPSS pilot study’s multiple data platforms – the cross-sectional outlet exit surveys with observation and verification of medicines and audits of all retail outlets and public health facilities - confirm that the subsidy of ACT increases access through the private sector to these life-saving medicines.

## Competing interests

AOT, PGD, AB, TE, BP, RC, ML, GB, JB and JG were at the time of the study affiliated with MMV a product development partnership-PDP for different anti-malaria drugs. KN, KR, ND work for Surgipharm Pharmaceuticals, which at the time of the pilot study was the company designated to distribute Coartem in Uganda. All the other authors declare no competing interest.

## Authors’ contributions

The members of the Consortium for ACT Private Sector Subsidy-CAPSS are the designated authors of this paper: AOT and PG (MMV) conceptualized the CAPSS pilot study, were the overall leaders of the management team and drafted the manuscript; JB, AB, GB, ML, RC and JG (MMV) were involved in the design and implementation of the pilot study and reviewed the manuscript; GBR, RN, MB, PK, FS and FK (Ug-MoH) were involved in the design and implementation of the CAPSS study and reviewed the manuscript; DN and AB (NDA) were involved in the regulatory aspects of CAPSS, training on safety monitoring and reviewed the manuscript; SMM, PB, JL, and AB (PACE) designed and implemented the secondary packaging and communications programme and reviewed the manuscript; GN and JT (MC) were responsible for the design and implementation of the training programme; KN, KR and ND (Surgipharm Pharmaceuticals) were involved in the design and management of the supply aspects of CAPSS; SR (I+Solutions) was involved in supply side and logistics training, SK (Management Sciences for Health-MSH) was involved in the design of the study and policy oversight and reviewed the manuscript; BP and TE (MMV) provided substantial input in the implementation of the field surveys and performed the geographical information system (GIS) and statistical analysis and reviewed the manuscript. All authors read and approved the final manuscript.
